# Erectile Dysfunction and Peyronie's Disease Following a Severe Case of COVID-19: A Case Report

**DOI:** 10.1155/criu/4329786

**Published:** 2025-07-04

**Authors:** Eric Qualkenbush, Evan Gibbs, Bryce Baird, Gregory Broderick

**Affiliations:** Department of Urology, Mayo Clinic Florida, Jacksonville, Florida, USA

**Keywords:** COVID-19, erectile dysfunction, penile duplex ultrasonography, Peyronie's disease, vasculopathy

## Abstract

The COVID-19 pandemic affected millions worldwide. While mainly regarded as a respiratory process, there may be both direct and indirect urologic consequences that are sparsely discussed. This case report describes a patient with a severe COVID-19 infection resulting in a kidney and liver transplant. After recovery, the patient was found to have de novo erectile dysfunction and Peyronie's disease. We suspect the systemic inflammation and vasculopathy leading to liver and renal failure also caused severe atherosclerotic erectile dysfunction. Furthermore, the prone positioning likely represents an iatrogenic etiology of Peyronie's disease. This unique case demonstrates some important considerations from a men's health perspective when caring for patients following a severe COVID-19 infection.

## 1. Introduction

The COVID-19 pandemic is estimated to have caused close to seven million hospitalizations in the United States alone [1]. The systemic inflammation seen in severe cases has been linked to atherosclerosis and vasculopathy [2–4]. Other works have found patients can have some degree of erectile dysfunction (ED) after a COVID-19 infection, though the exact mechanism is unclear [5, 6]. In this case report, we present a patient who, after a severe COVID-19 infection, was met with significant quality of life changes with new onset ED and Peyronie's disease (PD).

## 2. Case Presentation

A 44-year-old male was relatively healthy until he was hospitalized with COVID-19 in January 2022. His infection was complicated by severe shock and acute respiratory distress syndrome (ARDS) eventually requiring extracorporeal membrane oxygenation and prone positioning. His systemic disease resulted in multiorgan failure, culminating in a combination liver and renal transplant. Fortunately, he recovered and was discharged in stable condition.

He originally presented to our urology clinic where he endorsed intermittent penile pain and new onset ED. This began immediately after he was extubated in the ICU and felt extreme penile pain. The patient attributed this to being proned for 8 h daily and recalled being told that penile pain is common in proned patients by his intensive care team. Finally, upon discharge, the patient's penile pain began improving but was accompanied by new onset ED. He also found a slight curvature which continued to worsen significantly until the time of his presentation at our clinic.

**Figure 1:**
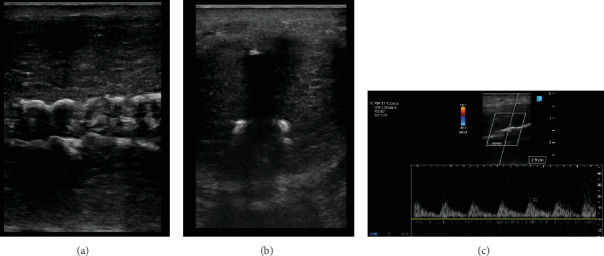
Penile ultrasonography. (a) Sagittal imaging with severe cavernosal calcifications. (b) Transverse imaging revealing shadowing from Peyronie's plaque and cavernosal calcifications. (c) Duplex ultrasonography showing poor peak systolic velocity of 17.10 cm/s.

**Figure 2:**
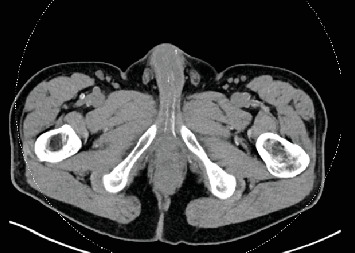
Cross-sectional imaging at the time of urology consultation with significant calcifications of the cavernosal artery bilaterally.

On physical exam, the patient was well developed with well healed surgical incisions. His penile shaft had palpable plaques on the mid dorsal aspect. This was accompanied by a dorsal bend of 45°–60° and was nontender to palpation. The Doppler penile ultrasound showed bilateral severe cavernous arterial insufficiency with transmural calcifications in the cavernous arteries (Figure 1). The cavernosal calcifications are grossly visible on his most recent cross-sectional imaging (Figure 2) and were not present on imaging during the early phases of his hospitalization. After discussing the findings and natural disease course with the patient, he elected to pursue penile traction therapy for his PD and daily tadalafil. At his 6-month follow-up, he no longer had penile pain and endorsed some improvement in his curvature. However, he still had ED and was offered penile prosthesis placement with modeling versus trialing an alternative phosphodiesterase inhibitor. To date, the patient has elected to continue conservative management and deferred any further operations.

## 3. Discussion

This case illustrates some significant long-term quality of life implications that are likely underdiscussed in patients following a severe COVID-19 infection with ARDS. Vasculopathy has been well documented to involve all major organ systems. We suspect it played a significant role in developing calcifications of his cavernosal arteries. This patient did not have a preexisting vascular disease seen on CT scans during the early parts of his hospitalization, yet imaging obtained less than a year later showed significant calcifications in his iliac and cavernous vessels. It is impossible to know if the calcifications seen here were directly caused by the systemic inflammation from COVID-19 or if this was sequelae from his short stint on dialysis, but nevertheless, the downstream effects of his respiratory illness ultimately impacted his erectile function.

In regard to his PD, there is one case report associating it to COVID-19, but no definitive evidence [7]. We suspect if the PD was not a direct result of COVID-19; then, this may demonstrate an indirect, iatrogenic cause of PD previously undescribed in the literature. The physiological effects of prone positioning can be lifesaving for patients with ARDS, but completing this maneuver is technically difficult and requires an attentive team. There is some thought that the penis is subjected to microtrauma when prone [8], and as such, this case demonstrates an instance of unavoidable stress on the penis. No guidelines exist for long-term penile accommodations while prone. This presents a future prospect for research and improvement. To our knowledge, this is the only described case of possible urologic trauma related to prone positioning.

The underdiagnosis of PD [9] and associations of ED with COVID-19 [10] illustrate the need to monitor patients' erectile quality following recovery. This patient survived ARDS with multiorgan failure and was able to resume living a normal day to day life but was left with significant quality of life implications with vascular ED and PD. There are over seven million COVID-19 related hospitalizations, and it is possible there are many other men suffering the long-term genitourinary sequelae from this global pandemic.

## Data Availability

The data that support the findings of this study are available on request from the corresponding author. The data are not publicly available due to privacy or ethical restrictions.

## Nomenclature

ARDS: acute respiratory distress syndrome
ED: erectile dysfunction
PD: Peyronie's disease
